# Premating Reproductive Barriers between Hybridising Cricket Species Differing in Their Degree of Polyandry

**DOI:** 10.1371/journal.pone.0019531

**Published:** 2011-05-05

**Authors:** Thor Veen, Joseph Faulks, Rolando Rodríguez-Muñoz, Tom Tregenza

**Affiliations:** 1 Centre for Ecology and Conservation, College of Life and Environmental Sciences, University of Exeter, Penryn, Cornwall, United Kingdom; 2 Biodiversity Research Centre, University of British Columbia, Vancouver, British Columbia, Canada; University of Texas Arlington, United States of America

## Abstract

Understanding speciation hinges on understanding how reproductive barriers arise between incompletely isolated populations. Despite their crucial role in speciation, prezygotic barriers are relatively poorly understood and hard to predict. We use two closely related cricket species, *Gryllus bimaculatus* and *G. campestris*, to experimentally investigate premating barriers during three sequential mate choice steps. Furthermore, we experimentally show a significant difference in polyandry levels between the two species and subsequently test the hypothesis that females of the more polyandrous species, *G. bimaculatus,* will be less discriminating against heterospecific males and hence hybridise more readily. During close-range mating behaviour experiments, males showed relatively weak species discrimination but females discriminated very strongly. In line with our predictions, this discrimination is asymmetric, with the more polyandrous *G. bimaculatus* mating heterospecifically and *G. campestris* females never mating heterospecifically. Our study shows clear differences in the strength of reproductive isolation during the mate choice process depending on sex and species, which may have important consequences for the evolution of reproductive barriers.

## Introduction

To understand speciation we need to elucidate the processes reducing gene flow between incompletely isolated populations of individuals. These processes lead to reproductive barriers, which come in many forms and may act at different times in the life of an individual and across different generations. Postzygotic barriers, those acting after fertilisation, have received most attention (e.g., [1]). A well-known example is genetic incompatibility between individuals from divergent populations leading to reduced fitness of hybrids [2]. These barriers are important in speciation and can be very strong (reviewed in [1], [3]), but are believed to only rarely act alone [4]. Prezygotic barriers can be seen as the ‘first line of defence’ against gene flow when two species come into secondary contact. These barriers can arise in allopatry (e.g., due to drift or adaptive changes in mate choice behaviours resulting from environmental differences) and become apparent in areas of secondary contact. Prezygotic barriers may also evolve or strengthen after secondary contact in response to hybridisation if this entails a cost (e.g., reduced hybrid fitness). In this latter case, known as reinforcement (reviewed in [5]), postzygotic costs drive prezygotic isolation.

Prezygotic barriers have been described between many different taxa, such as plants (e.g., [6], [7]), amphibians (e.g., [8], [9]), fishes (e.g., [10], [11]), insects (e.g., [12], [13]) and birds (reviewed in [14]). Gene flow during speciation may be restricted by a variety of different mechanisms. Obvious examples are mismatches in courtship song [15] and differences in timing of breeding [16]. But they may also be less obvious and arise from differences in life-histories between the species [17] or a variety of (interacting) ecological factors (reviewed in [1]). Lastly, gene flow may be impeded after copulation but before fertilisation by cryptic female choice processes such as conspecific sperm precedence [18], [19]. Besides the complication of many different processes potentially influencing prezygotic reproductive barriers, the detection of these barriers may depend on the spatial scale of the study [20]. In contrast to the many studies revealing the widespread occurrence of prezygotic barriers and their diversity, relatively few studies have investigated these barriers in great detail, checking for the occurrence of different barriers throughout the mate choice process and assessing each individual contribution to the overall barrier (but see [7], [8], [12], [21], [22]). Such detailed knowledge is invaluable for understanding the speciation process [4], [23]. The first aim of this study is to address this need for detailed studies of reproductive barriers by experimentally measuring the premating barriers between two closely related species of field crickets, *Gryllus bimaculatus* and *G. campestris,* during three sequential phases of mate choice.

Prezygotic reproductive barriers are hard to predict *a priori* due to the many different ways they may arise. For example, song may contribute towards species assortative mating if male signals differ between the species and female preferences match these differences, but it may have no effect if females of both species have open-ended preferences for the same signal characteristics [24]. An exception where the outcome may be more predictable might be hybridising species that differ in polyandry level. High levels of polyandry typically increase sexual conflict and drive evolution of male and female reproductive morphology and physiology [25]. These differences in turn are predicted to strengthen reproductive barriers between the species and hence increase speciation rates in clades of more polyandrous species [26], [27]. Polyandry, through sexual conflict, may also affect the strength of female preferences and hence their propensity to mate heterospecifically [27], [28]. If mating costs are higher in one species compared to the other, e.g., due to higher mate search costs, one would expect females of this species to mate with fewer males on average and be more selective in accepting a partner. However, polyandry creates the potential for females to exercise mate choice after copulation and hence more polyandrous species may be expected to shift some degree of female mate choice from precopulatory to postcopulatory processes [29]. Lastly, Gavrilets and Hayashi [30] put forward the idea that whether or not female choice evolves is the outcome of which sex ‘wins’ the sexual conflict: no choice if males win, and choice if females win. In all these scenarios the strength of preference is predicted to be lower in the more polyandrous species, which as a consequence, would be more prone to accept a heterospecific mate. This in turn may result in asymmetries in reproductive barriers between the two species, a widespread pattern found in nature [31].

The second aim of our study is to investigate the relationship between levels of polyandry, the strength of female mating preferences and asymmetric premating reproductive barriers. The effect of polyandry on the strength of female preferences has received little attention and its effects may vary among species. *G. bimaculatus* and *G. campestris* are well suited for our experiments as they are known to hybridise [32], have partially overlapping ranges [33], [34] and their mating behaviour is well-studied (e.g., [35], [36], [37]). Earlier studies in the wild showed that both species are polyandrous, and furthermore suggested that *G. bimaculatus* females mated most frequently and with more males [38], [39]. There is evidence that multiple mating in *G. bimaculatus* provides benefits to females because it allows them to exercise postcopulatory mate choice among partners rather than because of benefits of matings *per se* [40], [41], [42]. *G. campestris* has received less attention than *G. bimaculatus* but appear to have a similar mating system ([27], [43], RRM and TT personal observation). Females mount males prior to mating in both species, so we can exclude the idea as outlined above that multiple mating resulted from the males ‘winning’ sexual conflict. Furthermore, there is nothing to suggest that there are any differences in possible direct benefits between the species, which have similarly sized small spermatophores. We therefore hypothesise that the more polyandrous *G. bimaculatus* should show less strong mating preferences and an increased probability to mate heterospecifically. As our hypothesis implicitly assumes that the two species differ in levels of polyandry, we first experimentally determined the mating frequency and the number of different partners for females of both species.

To examine premating reproductive barriers between the two species, we conducted mate choice experiments involving both con- and heterospecific partners. The males produce a calling song to attract females to their location. Once a female has been attracted, the male switches to a distinct courtship song and initiates courtship behaviour, which involves presenting its abdomen to the female and trying to persuade her to mount and mate. Females of both species decide whether or not to mate (e.g., [44], [45]). Hybridisation between the two species has been identified in earlier, descriptive papers [32], [46]. von Hörmann-Heck [32] showed that *G. bimaculatus* females readily hybridised with a heterospecific male, but this was not the case for *G. campestris* females. We first validate these observations and extend them using an experimental approach by investigating in detail the response of males and females of both species to conspecific and heterospecific stimuli during the last stage of the mate choice process and quantify the strength of the reproductive barriers during its sequential stages. We address the following two questions: 1) how does mate choice create a reproductive barrier between *G. bimaculatus* and *G. campestris*, and 2) is the more polyandrous *G. bimaculatus* more likely to hybridise?

## Materials and Methods

### Ethics Statement

All experimental work complied with all relevant national and international guidelines.

### Study populations

For the mate choice experiments we used *G. bimaculatus* crickets from a lab-reared population originating from the Mediterranean coast near Valencia, Spain. For the polyandry experiment, first generation crickets from wild-caught individuals were used, captured in 2005. Individuals of both sexes were separated during their last instar and housed in individual boxes once adult. *G. campestris* were more difficult to breed in the lab for multiple generations and crickets were therefore specifically collected for the different experiments close to Oviedo, northern Spain (in 2006 and 2009/2010 for the polyandry and mate choice experiments respectively). Both wild-caught and lab-reared individuals were used for both experiments (see below). Both species were kept at an 18∶6 hour light:dark photoperiod at 25±1°C for the polyandry experiment and 16∶8 hour light:dark photoperiod at 28±1°C for the mate choice experiment, and given *ad libitum* standard rodent diet and water. The distributions of both species overlap in Spain, but the individuals used in this study originate from areas where only one of both species is living at present. *G. campestris* was not found within an area of about 40 km in diameter around the *G. bimaculatus* collection site, and the closest population of *G. bimaculatus* is at least 60 km away from the *G. campestris* collecting site, with the Cantabrian Mountains likely acting as a barrier between both. We therefore assume that the crickets used come from allopatric populations. Adults were reproductively mature (e.g., only males with a spermatophore present were used).

### Estimating mating frequencies and polyandry levels

F1-generation males and females of wild-caught *G. bimaculatus* and *G. campestris* nymphs collected in the field were housed in separate boxes in the lab. All boxes were checked daily for newly enclosed adults, which subsequently were transferred to individual boxes. The experiment was conducted in 2005 for *G. bimaculatus* and 2006 with *G. campestris*. Each experimental trial consisted of four females and four males being put in a large plastic box (0.75 m long ×0.50 m wide ×0.30 m high) with *ad libitum* food and water and eight shelters made out of corrugated tubing (mating was not possible inside the tube) for a period of four days. All activity inside each box was continuously recorded using four infrared cameras (using AnyKeeper v. 2516 (TechData Co., Ltd) for *G. bimaculatus* and Diginet Site v. 4.110 (Kodicom Co., Ltd http://www.kodicom.com/) for *G. campestris*). Only unrelated individuals (as far as we could control this) were used for the same trial and all females were virgin. Males were mated once to ensure that they were capable of mating. Each cricket was tagged using a unique combination of up to two white dots on their pronotum using correcting fluid. Boxes were cleaned prior to each experiment. The first 12 hours of video recordings of each trial were used to obtain the mating frequency (total number of mating events recoded) and the level of polyandry (number of unique partners mated with) for each female.

### Mate choice experiment

In the wild, after females locate and approach a male in response to his calling song, the two individuals make physical contact and start courtship. We simulated this situation by putting a male and female in a circular plastic container (0.145 or 0.17 m diameter) separated from one another by a barrier for at least 60 seconds. Time recording started when the crickets made physical contact. We subsequently recorded whether or not the male started its courtship song (which was the first step of the mate choice process in all but one case), the female mounted the male and if they successfully mated. The pair was continuously monitored for 10 minutes. A substantial proportion of the trials were monitored for one hour and the results showed only small quantitative differences compared to the first 10 minutes. This experiment was conducted using two different set-ups.

In the first experiment we used wild-caught *G. campestris* individuals of unknown age and mating status (virgin or not) collected in 2009 and 2010 at the same location and lab-reared *G. bimaculatus*. To maximise the number of mating trials, 17 of 87 females and 35 of 86 males *G. campestris,* and 18 of 72 females and 14 of 52 males *G. bimaculatus* were used multiple times, mostly twice (85%).

In a second experiment we used lab-reared *G. bimaculatus* and lab-reared offspring from wild caught *G. campestris* to control for factors such as differences in multiple use of the same individual, rearing condition or effects of the order of presenting conspecific / heterospecific partners [47]. The same protocol was used as for the first experiment, but each individual was used twice. First, 8 individuals were selected: two females and two males of each species. For the first trial (and for both species), one female was paired with a conspecific male and one with a heterospecific male. For the second trial, each female was confronted with a male of whichever species she had not encountered in the first trial. Thus each female was tested against a conspecific and heterospecific male in subsequent trials. Similarly, males were confronted with females of both species. A total of 8 sets of 8 individuals were tested. If mated, the spermatophore was removed after one hour. All individuals had a recovery period between trials of at least one hour (either when the experiment finished after 10 minutes without a mating or when it did after the spermatophore was manually removed). The containers were cleaned with ethanol before each trial to remove any odours and all experiment were conducted at 28±1°C.

### Statistical analyses

The mating frequency and the number of mates per female results were not normally distributed. We therefore used a Mann-Whitney U test to assess species differences. R was used for all analyses [48].

The close-range mate choice experiment results were analysed following the same sequential steps as outlined earlier; male courtship song, female mounting and whether this resulted in a successful mating (spermatophore transfer within 10 minutes). As the response variables are binary, we used a generalised-linear mixed model with a binomial distribution (glmm; R lme4 library). To control for the use of the same individual multiple times, an individual's ID (a unique number for each individual) was added as random factor. Only one random factor could be added (otherwise the size of the random effects vector exceeds the number of observations) which was chosen to be the ID of the sex that instigated the behavioural response (the male for the song, and female for all other behaviours). As explanatory variables we included; ‘heterospecific’ (whether or not the partner species was a heterospecific individual), species (*G. campestris*/ *G. bimaculatus*) and an interaction between the latter two to the model. The experiments with the wild-caught and the first generation lab-reared *G. campestris* were analysed separately. The decision to exclude an explanatory variable from the model was based on whether or not model fit was significantly reduced. This was tested using ANOVAs with Chi^2^ distribution [49]. In the presentation of the results of statistical tests, we presented the ANOVA output of the model reduction steps (so whether or not the variable would significantly reduce model fit if excluded) and the z value of the glmm. All variables retained in the final model are presented in bold. Means in the text are presented with standard deviations in brackets and where required controlled for multiple measurements by taking the average for each individual (sample size thus represent number of individuals used, not number of trials).

## Results

### Mating frequencies and polyandry levels

Five trials were conducted for *G. bimaculatus* and 9 for *G. campestris*, totalling 19 and 36 individual females respectively (one female *G. bimaculatus* died during the experiment). *G. campestris* were all of a similar age (11.9 (1.4) days) but there was considerably more variation in *G. bimaculatus* (23.5 (13.2) days). However, as there was no significant correlation between age and number of matings nor number of males (linear regression for number of matings; F_1, 17_ = 0.016, p = 0.902, and number of males mated with; F_1, 17_ = 0.959, p = 0.341) we left age out of the further analyses. *G. bimaculatus* females mated significantly more often during the 12 hour period compared to *G. campestris* (8.7 (5.4) and 1.6 (1.4) times, respectively; Mann-Whitney U test W = 640, p<0.001). The level of polyandry was significantly higher for *G. bimaculatus* females (3.3 (0.8) males) compared to *G. campestris* females (1.3 (0.9) males; Mann-Whitney U test W = 642, p<0.001).

### Mate choice experiments

#### Wild-caught *G. campestris* and lab-reared *G. bimaculatus*

**Figure 1:**
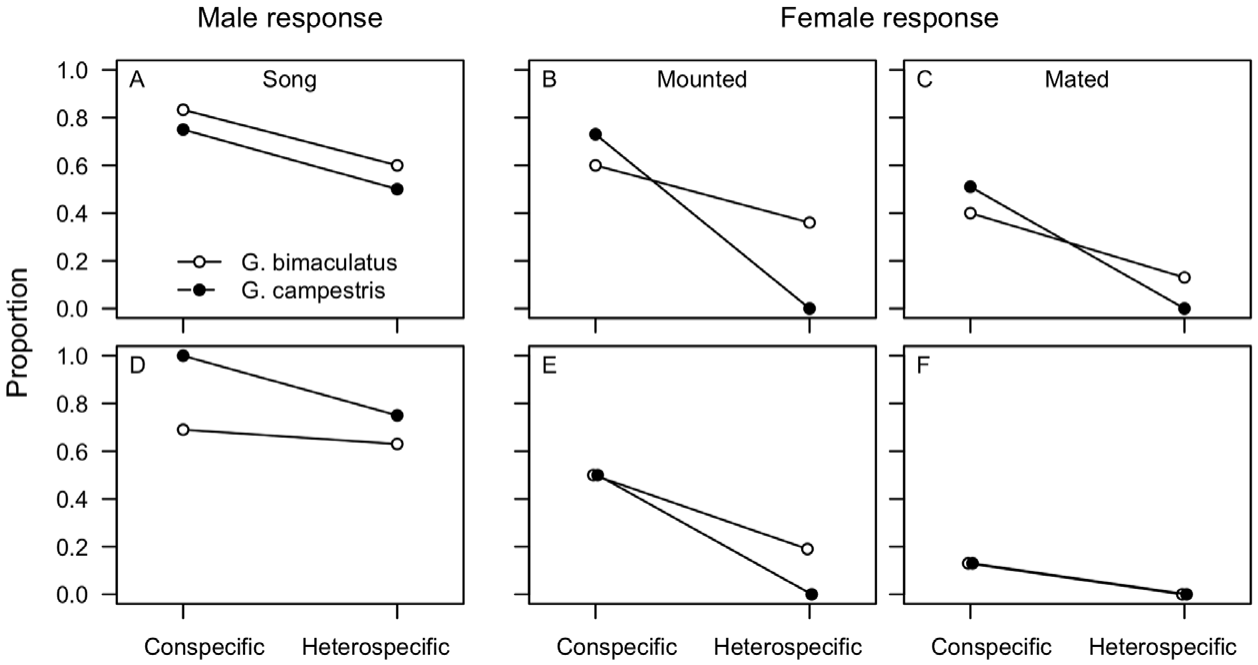
Mate choice experiments. Males and females of the two species were paired in con- and heterospecific pairs and the proportion of males starting courtship song (**A**, **D**), females mounted (**B**, **E**) and mated after 10 minutes (**C**, **F**). The top row (**A**–**C**) represents trials using wild-caught *G. campestri*s (2009 and 2010) of unknown age and mating status. The captive bred offspring of the wild-caught *G. campestri*s (2009) were used in a subsequent mate choice experiment (**D**–**F**) in which e.g., rearing condition, mating status and mating sequence could be controlled for (see main text for more details). Lab-reared *G. bimaculatus* were used for all trials.

The experiment involved 48 *G. bimaculatus* (female) x *G. bimaculatus* (male), 84 *G. campestris* x *G. campestris*, 45 *G. bimaculatus* x *G. campestris* and 20 *G. campestris* x *G. bimaculatus* mate choice trials. A large proportion of males started singing within the first 10 minutes when paired with a conspecific female (0.79), but significantly fewer with a heterospecific female (0.55; Figure 1A and Table 1). The non-significant interaction indicated that there was no difference in the strength of males' response towards conspecific or heterospecific females (Table 1). From a biological perspective it is valuable to note that at least half of the males did sing towards heterospecific females.

**Table 1:** Mate choice experiment using wild-caught *G. campestris* and lab-reared *G. bimaculatus*.

			glmm	model selection		
response variables	random effect	explanatory variables	z	df	Chi^2^	p
song	male ID	**intercept**	6.288			
		male species (MS)	−0.587	1,4	0.332	0.565
		**heterospecific (H)**	−3.183	1,3	9.347	0.002
		MS × H	1.159	1,5	1.263	0.261
mounting	female ID	**intercept**	1.493			
		**female species (FS)**	1.527			
		**heterospecific (H)**	−2.530			
		**FS** × **H**	−0.012	1,5	16.385	<0.001
mated	female ID	**intercept**	−1.444			
		**female species (FS)**	1.296			
		**heterospecific (H)**	−2.741			
		**FS** × **H**	−0.010	1,5	6.030	0.014

Generalised linear-mixed model analyses looking at the propensity of males to start singing, and the propensity of females to mount and mate within 10 minutes to con- and heterospecific partners (see main text for details). The *G. campestris* used in this experiment were wild-caught in 2009 and 2010. The *G. bimaculatus* were raised in the lab. Explanatory variables retained in the final model are in bold.

Females of both species mounted heterospecific males (0.18) less often compared to conspecific males (0.67). Female *G. campestris* were much more inclined to mount conspecific and not heterospecific males compared to *G. bimaculatus* females, as indicated by the significant heterospecific × female species interaction (Figure 1B and Table 1). Similarly, females of both species mated with conspecific males (0.45) more readily compared to heterospecific males (0.07) and again the difference in propensity to mate with a conspecific over a heterospecific male was significantly stronger for *G. campestris* females (Figure 1C and Table 1).

To validate the robustness of these results with respect to multiple use of the same individual, we reanalysed the data but only allowing each individual to be used in different pairing types (pure and mixed-species trials) and tested on different days (n = 154 trials). Qualitatively, the results were very similar but the heterospecific × female species interaction for mating was not significant (p = 0.076) and only heterospecific was retained in the final model.

#### Lab-reared *G. campestris* and *G. bimaculatus*

A total of 64 individuals (8 sets of 8 individuals) were used in the second experiment. The males of the two species differed significantly in the propensity to sing in response to a conspecific (0.84) compared to a heterospecific female (0.69) and *G. campestris* males did this significantly more frequently (Table 2 and Figure 1D).

**Table 2:** Mate choice experiment using only lab-reared crickets.

			glmm	model selection		
response variables	random effect	explanatory variables	z	df	Chi^2^	p
song	male ID	**intercept**	1.287			
		**male species (MS)**	0.000			
		**heterospecific (H)**	0.990			
		**MS** × **H**	0.000	1,5	12.879	<0.001
mounting	female ID	**intercept**	0.002			
		female species (FS)	−3.324	1,4	0.805	0.370
		**heterospecific (H)**	−3.321	1,3	13.775	<0.001
		FS × H	−0.006	1,5	3.632	0.057
mated	female ID	**intercept**	−0.552			
		female species (FS)	−0.023	1,4	0.053	0.818
		**heterospecific (H)**	−0.402	1,3	28.010	<0.001
		FS × H	0.000	1,5	0.051	0.822

Generalised linear-mixed model analyses looking at the propensity of males to start singing, and the propensity of females to mount and mate within 10 minutes to con- and heterospecific partners. The experimental set-up between the mate choice tests differed slightly. In this experiment each individual was tested against both a hetero- and conspecific partner and we controlled for presentation order (see main text for details). The *G. campestris* used in this experiment were the F1-generation from wild-caught individuals and reared in the lab in 2009. The *G. bimaculatus* were raised in the lab for several generations. Explanatory variables retained in the final model are in bold.

Females of both species mounted conspecific males much more readily than heterospecific males (0.50 and 0.09, respectively; Figure 1E and Table 2) and this effect was strongest for *G. bimaculatus,* although not significantly so (p = 0.057). Females mated significantly less frequently with heterospecific males (0) compared to conspecific males (0.13) after 10 minutes (Figure 1F and Table 2). This pattern changed a little after one hour. Although both *G. bimaculatus* and *G. campestris* females mated more readily with conspecifics (0.38 and 0.19, respectively), only *G. bimaculatus* was found to hybridise (0.13).

## Discussion

Our results show a strong reproductive barrier between *G. bimaculatus* and *G. campestris*. Both species preferred conspecific partners, but *G. bimaculatus* females were less discriminating and did mate heterospecifically (which was never recorded for *G. campestris* females). This asymmetry in hybridisation frequency is in line with an earlier study [32]. Furthermore we found a significantly higher mating rate and polyandry level for *G. bimaculatus* females and together with the asymmetric reproductive barrier this supports the hypothesis that polyandry levels are positively correlated with the propensity to mate heterospecifically.

Our mate choice experiments revealed premating barriers between the two species arising during both the male and female controlled aspects of courtship and mating. The first barrier was caused by males of both species starting their courtship song more readily in response to conspecific females. The second barrier was very strong and resulted from females of both species being more reluctant to mount and mate with heterospecific males (*G. campestris* never mounted a heterospecific male). Our results confirm von Hörmann-Heck's [32] strong asymmetric barrier between the two species (although *G. campestris* females did hybridise very rarely (only twice) during her trials). We did not find a strong asymmetry between the species with respect to which sex is most choosy, which contrast with claims made by von Hörmann-Heck. She states that males made the choice in *G. bimaculatus* but females in *G. campestris* due to differences in sex recognition between the species (although we find the reasoning behind this conclusion hard to follow). We believe our mating data from the only lab-reared individuals experiment to be more accurate, as this controlled for more variables and followed the multiple mate choice stages in greater detail.

The results from the two different experiments (using wild-caught or lab-reared individuals) were very similar, especially for the strongest, female induced reproductive barriers, which strengthen our conclusions.

Although we show the existence of two reproductive barriers, we can only speculate what the reasons for these barriers are as we did not manipulate sexual signals in this experiment. One possibility is that the species use different or multiple signals at close-range [21], [32], [50]. Cuticular hydrocarbons are a good candidate, as these are known to be used by crickets and differ between the two species (unpublished data, see also [51]).

Based on our experiments we predict that gene flow should be significantly reduced in the contact zone between the two species. However, there are several potentially important factors influencing mating patterns in nature. The relative abundance of the two species in the wild influences the likelihood of encounters between conspecific mates and this will affect realised mating frequencies [31]. However, our results show that patterns of asymmetric hybridisation may also be caused by other mechanisms than skews in species abundance. Furthermore, we compared an individual's response to stimuli (con- and heterospecific) from only two individuals (with the exception of some individuals in the ‘wild-caught’ trials). Potentially the strength of species differentiation may be affected by exposure to (conspecific) sexual signals [14], [47]. Finally, it is important to realise that mixed-species pairing does not need to result in higher rates of hybridisation (i.e. the production of hybrid offspring) due to conspecific sperm precedence or other processes [18], [52], [53] and hence may not increase gene flow between the species.

Prezygotic reproductive barriers have been described in many different taxa and crickets are no exception [12], [51], [54], [55]. The results from these studies and ours are broadly similar, but differ on several important points. Other studies also found differences between sexes and species during close-range mating behaviour [51], [54], [56]. Females are frequently found to prefer conspecific males over heterospecific males (e.g., [12]), but this is not always the case [21], [47]. The weaker reproductive barrier during male initiated courtship is found in at least one other study [47]. Species differences in propensity to mate heterospecifically resulting in unidirectional hybridisation appear to be relatively common [12], [31], but completely asymmetrical reproductive barriers as found in this study (*G. campestris* never mated with *G. bimaculatus* but see [32]) appear to be rare (see [57] for an exception). Our study does not allow us to determine which mechanisms underlie the reproductive barriers and this can be a set of different factors, such as differences in song [58], [59] and cuticular hydrocarbons [60]).

Several other premating barriers besides those during close range mate choice (as investigated in this study) have been found between hybridising crickets species. Temporal isolation may arise in at least parts of range between *G. pennsylvanicus* and *G. firmus* due to differences in emergence time of nymphs [16]. During the initial stages of mate attraction over longer ranges, females have been found to prefer conspecific calling songs, but the strength of this barrier is variable ([54], [55], [58] but see [56]).

The diversity of differences in strengths of prezygotic reproductive barriers between the different sexes, species, and when they act between different closely related (*Gryllus*) cricket species is remarkable. This might lead to different adaptive responses (as indicated by [46]), making this group of insects a particularly promising one to study speciation.

An important aim of this paper was to attempt to use prior knowledge of the mating system of both cricket species to predict the direction of the reproductive barriers. We first quantified the difference in polyandry levels between the two species and found a significant difference, although not very large. Note though that this estimate is most likely conservative, as the maximum number of potential unique mates was four in this experiment.

Our prediction that the more polyandrous *G. bimaculatus* is more likely to mate heterospecifically is supported by our results. Polyandry in *G. bimaculatus* has been shown to have the potential to decrease inbreeding [40], [42] and to allow selection for genetically more compatible mates [41]. This supports the idea that female choice may have switched towards postcopulatory processes and that this may influence the strength and symmetry of premating reproductive barriers. Potentially, differences in mate search cost or in levels of female cryptic choice may be important but empirical data are lacking. Other differences between the species, or even within species [61], not related to the level of polyandry, may lead to differences in choosiness and the pattern of hybridisation found. One such alternative is that *G. campestris* females differ in their sexual signals used or use additional sexual signals for mate choice and hence do not perceive heterospecific males as potential partners.

Lastly, selection pressures on multiple mating may differ between the sexes. A recent study [21] reports high costs of multiple mating for males (and none for females) and speculates that this could result in increased selection against heterospecific mating (as these have lowest fitness returns) in males. Although this suggestion is not supported by our results, we found males to be the less discriminating sex, it does stress the need of a holistic approach to understanding complex reproductive barriers.

Sexual conflict may lead to asymmetric hybridisation and the differences in rate of gene flow is predicted to differentially affect the standing genetic variation in both species, which in turn influences other evolutionary processes such as the capability to adjust to different environments [62]. At present there are too many gaps in our empirical and theoretical knowledge to say whether or not differences in mating systems play such a significant role in speciation (in crickets) and how large these differences need to be to have an effect. More empirical work is needed and should ideally focus on species with larger differences in polyandry levels than reported here.

Our study contributes to an increasing body of empirical evidence that reproductive barriers can be complex. We set out to quantify the premating reproductive barriers between *G. bimaculatus* and *G. campestris* and to test the hypothesis that the more polyandrous species is more prone to mate heterospecifically due to reduced female choosiness. Our results show reproductive barriers, which differed in magnitude depending on sex and species. The asymmetry found was in the predicted direction, but alternative explanations not linked with levels of polyandry cannot be excluded. The complexity of reproductive barriers indicates that the usual approach of conducting experiments that focus on only a single aspect of premating reproductive isolation may not be sufficient. This suggests a need for further theoretical work integrating these areas to guide future empirical work [30], [63] alongside empirical efforts to increase the small number of studies that quantify in detail reproductive barriers at more than one stage in the reproductive process [8].
